# Enhancing Team-Based Learning in Virtual Environments: The Role of Avatar Agency and Immersive Social Presence

**DOI:** 10.2196/92366

**Published:** 2026-03-30

**Authors:** Hiroki Funao, Motomu Shimaoka, Jun Kako

**Affiliations:** 1 Mie Prefectural College Of Nursing, 1-1-1 Yumegaoka, Tsu, Mie, 514-0116, Japan, 81 59-233-5600; 2 Department of Molecular Pathobiology and Cell Adhesion Biology, Graduate School of Medicine, Mie University, Tsu, Mie, Japan; 3 Department of Nursing, Graduate School of Medicine, Mie University, Tsu, Mie, Japan

**Keywords:** anesthesia, computer-assisted instruction, distance, education, internet, learning, medical, problem-based learning, students, teaching, virtual reality

We read with great interest the report by Sripadungkul et al [1] comparing web-based virtual environment (WBVE) with face-to-face (F2F) delivery for team-based learning (TBL) of anesthesia techniques. Their finding that WBVE-delivered TBL yields knowledge gains similar to those of F2F delivery while offering scalability is encouraging for medical education. However, their finding of decreased learner satisfaction levels in the WBVE group warrants further discussions of the role of avatar agency and immersion degree.

Sripadungkul et al [1] used a desktop-based WBVE on the Spatial platform and noted that the lack of “nonverbal cues” might have led to decreased learner satisfaction levels compared to F2F delivery. We argue that this decreased satisfaction is a limitation of the nonimmersive virtual environment using a 2D desktop interface rather than the virtual environment pedagogy itself. Recent evidence comparing head-mounted display (HMD)–based immersive 3D virtual reality (VR) with traditional F2F instruction demonstrates comparable or even superior results regarding student acceptance in medical education. Mühling et al [2] demonstrated that medical students rated HMD-based VR stations as having realism and clinical relevance equivalent to traditional physical objective structured clinical examination stations, with no significant differences in overall perception. Notably, the students exhibited high acceptance of the VR system, suggesting that active participation from a first-person perspective in an immersive environment positively influenced their engagement. In nursing education, research on novice nurses revealed that immersive VR training was more effective than conventional F2F group discussions in fostering significantly greater and sustained improvements in collaborative attitudes. This effectiveness likely stems from the realistic reproduction of interpersonal environments in an immersive VR space that prevents communication hindrances [3].

Our research on the feasibility of immersive VR-based group discussions among nursing students supports the importance of “avatar agency,” allowing users to subjectively and actively control and personalize their digital representations [4]. Over 70% of our study participants reported that personalizing avatar characteristics, including clothing/hairstyles, influenced their discussions, and all participants expressed a high level of intention to continue using VR for learning [4]. Entering an immersive virtual environment using avatars fosters “social presence,” allowing users to feel they are in the same space, encouraging interactive communication [5]. These elements minimize the “communicative gap” and could bridge the satisfaction gap identified by Sripadungkul et al [1]. There could also be increased motivation to engage in tasks and establish a virtual environment that serves as a foundation for effective TBL. Therefore, we would like to request further details on the avatar settings and authors’ perspectives.

Although the introduction of HMDs is critical to such enhancements, the implementation costs are high; therefore, careful consideration of their impact on scalability in large-scale educational settings is required. WBVE delivery of TBL is a scalable alternative to F2F delivery, integrating more immersive elements and allowing for greater avatar agency, which could improve its learner satisfaction rate. We appreciate the authors’ contribution to this evolving field and believe that exploring the interaction between immersion and social presence will help establish optimal practices for future virtual health care education.

## Abbreviations

F2F: face-to-face
HMD: head-mounted display
TBL: team-based learning
VR: virtual reality
WBVE: web-based virtual environment
